# Improved treatment of community-acquired pneumonia through tailored interventions: Results from a controlled, multicentre quality improvement project

**DOI:** 10.1371/journal.pone.0234308

**Published:** 2020-06-11

**Authors:** Markus Fally, Christian von Plessen, Jacob Anhøj, Thomas Benfield, Britta Tarp, Lise Notander Clausen, Lilian Kolte, Emma Diernaes, Line Molzen, Regitze Seerup, Simone Israelsen, Anne-Marie Blok Hellesøe, Pernille Ravn

**Affiliations:** 1 Department of Internal Medicine, Section for Pulmonary Diseases, Herlev Gentofte Hospital, Hellerup, Denmark; 2 Unisanté, Lausanne, Switzerland; 3 Department of Clinical Research, University of Southern Denmark, Odense, Denmark; 4 Centre of Diagnostic Investigation, Rigshospitalet, Copenhagen, Denmark; 5 Department of Infectious Diseases, Amager Hvidovre Hospital, Hvidovre, Denmark; 6 Diagnostic Centre, Silkeborg Regional Hospital, Silkeborg, Denmark; 7 Department of Respiratory and Infectious Diseases, Nordsjaellands Hospital, Hillerød, Denmark; 8 Department of Internal Medicine, Section for Infectious Diseases, Herlev Gentofte Hospital, Hellerup, Denmark; University of California San Diego, UNITED STATES

## Abstract

**Background:**

Community-acquired pneumonia (CAP) is one of the leading causes of healthcare utilisation and death worldwide. Treatment according to evidence-based clinical guidelines can reduce mortality, antibiotic exposure and length of hospital stay related to CAP.

**Local problem:**

Several studies, including a pilot study from one of our sites, indicate that physicians show a low grade of guideline adherence when managing patients with CAP.

**Methods:**

To improve the guideline-based treatment of patients with CAP admitted to hospital, we designed a quality improvement study. Four process indicators were combined in a CAP care bundle: chest X-ray, CURB-65 severity score, lower respiratory tract samples and antibiotics within 8 hours from admission. After a 4-month baseline period, we applied multiple interventions at three hospitals during 8 months. Progression in our process indicators was measured continuously and compared with a control site without interventions. After the 8-month intervention period, we continued with a 4-month follow-up period to assess the sustainability of the improvements.

**Results:**

The care bundle utilisation rate within 8 hours increased from 11% at baseline to 41% in the follow-up period at the intervention sites, whereas it remained below 3% at the control site. The most considerable improvements have been observed regarding documentation of CURB-65 (34% at baseline, 68% at follow-up) and the collection of lower respiratory tract samples (43% at baseline, 63% at follow-up).

**Conclusion:**

Our study has demonstrated poor adherence to CAP guidelines at all sites at baseline. After implementing multiple tailored interventions, guideline adherence increased substantially. In conclusion, we recommend that CAP guidelines should be actively adapted in order to be followed in a daily routine.

## Introduction

Community-acquired pneumonia (CAP) is a common disease and causes significant morbidity and mortality, particularly among the elderly and patients with comorbidity [1]. In Denmark, CAP accounts for approximately 45.000 hospitalisations annually, with a 30-day mortality of 10–15% [2,3].

In recent decades, professional societies have developed evidence-based guidelines to assist clinicians in treating patients with CAP [4–7]. Adherence to these guidelines can reduce antibiotic exposure, length of stay, mortality and health care costs without negatively affecting patient outcomes [8–11].

Generally, adherence to guidelines is highly variable and differs depending on the local context, disease and outcome of interest [12–14]. In line with these findings, unpublished data from a pilot study at a Danish regional hospital indicated low adherence to CAP guidelines, including the infrequent collection of microbiological samples and delayed and protracted use of antibiotics (Ravn et al., personal communication). Such unsatisfactory adherence to CAP guidelines can be attributed to numerous factors, including physicians’ knowledge, beliefs and preferences, and inefficient health care processes, as well as the heterogeneity of the manifestations of CAP [15].

One strategy to increase evidence-based patient management is the implementation of care bundles [16]. A systematic review on the general effect of care bundles concluded that they may reduce the risk of negative outcomes; however, the quality of evidence was very low [17].

The care bundle approach has been successful in the prevention of ventilator-associated pneumonia (VAP), reducing VAP incidence, length of stay and mortality [17–22]. Few studies have explored the effect of care bundles on the quality of care for CAP and those were not conclusive. One recently published study did not identify any outcome benefits of implementing a care bundle for CAP patients [23]. However, this may be due to the study’s small size and the inclusion of heterogeneous and controversial elements of CAP care, such as corticosteroid treatment [23]. Two larger studies with 2819 [24] and 23315 [25] patients, respectively, reported that the implementation of a CAP care bundle led to a higher proportion of patients receiving antibiotics within 4 hours as well as a reduction in mortality rate.

To enhance patient care in CAP, our project aimed to increase adherence to a care bundle at three clinical sites in Denmark. A fourth site served as a control site without any interventions.

## Methods

### Project design and local context

The Optimising Treatment of Community-Acquired Pneumonia (optiCAP) project was designed as a 16-month, prospective, open, interventional, controlled, multicentre quality improvement study, evaluating a change programme by applying statistical process control (SPC) [26].

The study was conducted at four regional hospitals in Denmark: Nordsjaellands Hospital (Site 1), Gentofte Hospital (Site 2), Silkeborg Regional Hospital (Site 3) and Hvidovre Hospital (Site 4). At each hospital, emergency departments (EDs), as well as departments of respiratory medicine and infectious diseases, served as study sites. S1 Fig in the S1 File presents an overview of the study sites, with further details available in S1 File.

Based on a baseline audit (November 2017 until February 2018), interventions were scheduled to take place from March until October 2018 at Sites 1–3. This included both technical and non-technical interventions based on the model for improvement, including Plan-Do-Study-Act (PDSA) cycles [27]. Site 4 served as control site without any interventions, and thus without deliberate attempts to improve the quality of CAP care.

During a follow-up period from November 2018 until February 2019, we evaluated the sustainability of the improvements.

### Study population

The present study included adult patients admitted to our study sites (age ≥ 18 years) with CAP, treated with antibiotics. The diagnostic criteria used to define CAP are generally very heterogeneous [28]. In our study, we used one of the common definitions. Hence, CAP was defined by the presence of a new infiltrate on chest X-ray and at least one of the following signs and symptoms: cough, sputum production, dyspnoea, core body temperature >38.0°C and auscultatory findings of rales.

Exclusion criteria were hospital admission during the last 28 days, active tuberculosis and immunosuppression. Patients were classified as immunosuppressed if they had received treatment with corticosteroids (≥20 mg prednisolone-equivalent/day > 14 days), were HIV positive, had received chemotherapy during the last 28 days, had neutropenia (< 1000/μl), were an organ transplant recipient or received biological response modifier therapy.

### Measures

Based on current evidence, patients admitted with CAP should be diagnosed and receive antibiotic treatment within 8 hours from admission, as this reduces mortality [29]. Furthermore, the collection of lower respiratory tract samples is recommended for all patients admitted with CAP in Denmark. As the Scandinavian countries continue to use narrow-spectrum antibiotics for most CAP patients, the microbiological results may eventually be used for altering the antibiotic therapy [4,30,31]. In Denmark, as in other countries, the CURB-65 score is used to assess CAP severity and ultimately to determine which type of empiric antibiotic to prescribe [4–6].

Based on these facts, we defined the following key indicators of adequate care for CAP:

- Process measures [27], i.e. actions completed within 8 hours after admission:
  1. Chest X-ray
  2. Collection of lower respiratory tract samples (LRTS)
  3. Documentation of mortality risk assessment by CURB-65 score (confusion, plasma urea, respiration frequency, blood pressure, age 65 or older) [32]
  4. Administration of an antibiotic
- Outcome measure [27]: Care-bundle treatment, i.e. proportion of patients who received all four elements as described in the previous point

### Data collection

At each site, data were collected weekly by auditing electronic health records. An audit of 5 to 10 health records per site per week was considered appropriate, and these numbers could be reached by including all CAP patients at out study sites [27]. Local teams collected the data, which were transferred and stored in Research Electronic Data Capture (REDCap, version 9.3) software [33]. Raw data were subsequently cleansed using tidyverse (version 1.2.1) for R (version 3.6.0, R Core Team 2019). This implicated the detection and correction of missing values, negative values and outliers (values below the first and above the third quartile).

### Baseline results and theory of change

Baseline data indicated variation in performance at the different sites, but also room for improvement regarding all of the defined process measures. Median time (interquartile range [IQR]) to chest X-ray was 2.3 (1.2–4.5) hours, corresponding to a completion rate of 88.7% within 8 hours. LRTS were collected in 60.6% of cases; the completion rate was 39.6% within 8 hours. CURB-65 score was documented in 31.2% of cases at a completion rate of 26.9% within 8 hours. The median time to administration of the first antibiotic (IQR) was 5.2 (3.4–8.0) hours, corresponding to a completion rate of 75.1% within 8 hours. The complete care bundle was delivered within 8 hours in 7.1% of cases.

Non-adherence to most of the given recommendations was an issue at all sites. To develop solutions and improve adherence to CAP guidelines, our team defined the following explanatory theories over the course of several seminars and meetings [34]:

- Lack of information regarding the disease as well as how and why to manage it as recommended by the guidelines
- Lack of skills regarding acquisition of LRTS
- Ineffective processes and unclear responsibilities
- Lack of effective electronic health record system (EHRS) tools to guide clinicians

Through the following approaches, we expected that we could reach our aim of achieving sustainable changes [34]:

- Provide more and easily accessible information on the disease, current guidelines and evidence-based practices, clearly explaining why they should be applied
- Provide feedback to clinicians when care delivery problems are discovered during the audit process
- Change ineffective processes using an integrative approach (feedback from clinicians)
- Streamline information and guideline content

**Fig 1:**
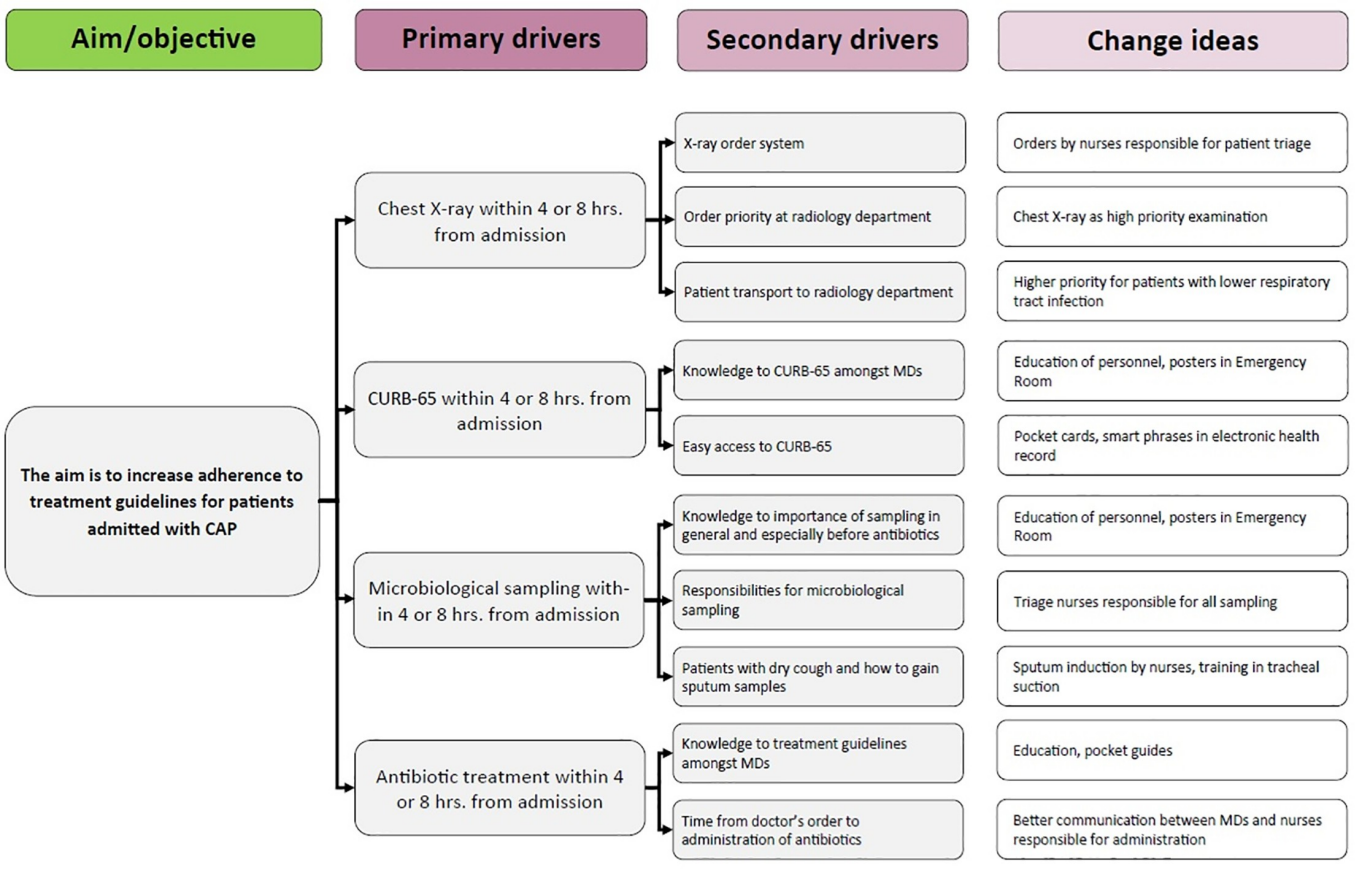
Drivers and detailed change ideas derived from our theory of change. CAP: community-acquired pneumonia, CURB-65: pneumonia mortality risk score (confusion, urea, respiration frequency, blood pressure, age 65 or older) [32], MD: medical doctors.

The drivers and detailed change ideas are provided in the driver diagram in Fig 1.

### Interventions

The interventions included both non-technical and technical activities (Table 1), which were derived after a baseline workflow assessment using optiCAP team members as observers. Furthermore, staff members were interviewed to identify limitations of the use of CAP guidelines and to gather suggestions for improvement.

**Table 1:** An overview of the implemented interventions.

		Site 1	Site 2	Site 3
**Technical interventions**				
**Educational activities**				
	Repeated hands-on training in tracheal suction for physicians		x	x
	Repeated hands-on training in tracheal suction for nurses	x		x
	Repeated hands-on training in sputum induction by nurses		x	
**Non-technical interventions**				
**Educational activities**				
	Repeated education of physicians at the relevant departments	x	x	x
	Repeated education of nurses at the relevant departments	x	x	x
	Personal face-to-face feedback to physicians			x
	Personal feedback to physicians via email		x	
	Personal feedback to physicians via the feedback option in the health record system			x
**Educational material**				
	Standardised PowerPoint presentations on CAP	x	x	x
	Pocket cards on CAP	x	x	x
	Regular newsletter distribution	x	x	x
	Posters on guideline-based CAP treatment at the departments		x	
**Process improvements**				
	Authorising triage nurses to order X-rays		x	
	Authorising triage nurses to order LRTS	x	x	
	MCS and PCR for atypical bacteria analysed using the same LRTS	x	x	
	CURB-65 as a standard phrase in the EHRS	x	x	x
	Order sets for CAP in the EHRS	x	x	x

Abbreviations: CAP: community-acquired pneumonia; LRTS: lower respiratory tract sample; MCS: microscopy, culture, sensitivity; PCR: polymerase chain reaction; EHRS: electronic health record system.

The technical interventions were designed to increase the amount of LRTS and consisted of hands-on training for physicians and nurses. Training in tracheal suction consisted of repeated sessions 10–20 minutes in duration with 5–15 participants. A senior physician explained and performed the procedure on a dummy. The training was offered to physicians (Sites 2 and 3) and nurses (Sites 1 and 3). After these sessions, participants were encouraged to continue with bed side training under the supervision of more experienced colleagues. At Site 2, an experienced senior nurse designed and implemented hands-on training in sputum induction, which consisted of a 5-10-minute theoretical introduction followed by bed-side training for 1–3 nurses per session.

Regarding the non-technical interventions, we divided the activities into (1) educational activities, (2) the design and distribution of educational material and (3) improvement of CAP patient flow.

The education of nurses and physicians involved repeated presentations lasting 15–45 minutes. Presentation topics included CAP pathogenesis, epidemiology, and guideline-based assessment and treatment. Later, during the intervention period, the results of the ongoing audit were presented and discussed. These educational sessions were held by an optiCAP team member, often as a part of the morning report or other regular teaching sessions involving 10–40 participants. Additionally, physicians received both confirming and corrective feedback from the study team during the health record audit. Feedback was always provided personally, either face-to-face, via secure work email or via the message system in the electronic health record system.

Educational material on the most important aspects of CAP included slide presentations for education sessions, newsletters to all physicians, and posters in all staff offices of the departments involved in the project, as well as pocket cards related to CAP. This material provided information on important steps in the assessment and treatment of CAP. The newsletters and presentations also included audit results and figures on the overall progress of the project.

Process changes involved authorising nurses to order X-rays and LRTS based on triage information at the ED, as well as an agreement with the Department of Clinical Microbiology to perform both standard microscopy, culture and sensitivity analysis (MCS) along with PCR for atypical bacteria from a single LRTS. Previously, this testing required two samples, and physicians often had to choose which test to perform. Furthermore, we developed several tools for the electronic health record systems (EHRS; Epic EMR and MidtEPJ) to assist physicians in treating CAP patients. These tools offered standard phrases for CURB-65 documentation as well as order sets consisting of guideline-based tests and antibiotic treatment packages.

### Study of interventions and data analysis

For effect evaluation, we produced run charts with qicharts2 (version 0.6.0) for R [35]. Run charts are point-and-line graphs used to distinguish random from special cause (non-random) variation in time series data. The R code to reproduce the run charts and the final database are attached in the Supporting Information. For interpretation, we applied the Anhoej rules with the median as process centre [36]. The Anhoej rules are two tests for special cause variation: (1) unusually long runs of consecutive data points on the same side of the centre line, and (2) unusually few crossings of the centre line. Critical values for run length and number of crossings depend on the number of available data points and may either be calculated or found in statistical tables [36–38]. For example, in a run chart with 24–26 data points, a run of more than 8 data points or fewer than 8 crossings would indicate the presence of special cause variation. In the initial analysis, we used the median from the baseline period, including data from 12 ten-day periods each representing 12–23 patients at the control centre and 31–48 at the intervention sites as reference. With 49 data points in total, special cause variation was declared if any run exceeded 9 data points in length or if the curve crossed the centre line fewer than 19 times. Next, we recalculated the process centre (medians) for four periods: the baseline period, early intervention period (March 2018 to July 2018), late intervention period (August 2018 to October 2018) and the follow-up period.

### Ethical considerations

The presented project was designed as a clinical audit and quality improvement project without direct patient contact. Therefore, the national authorities determined that ethical approval was not required to conduct this project. Local approval was granted by the respective hospital boards. Data for analysis were anonymized and handled according to the national regulations of the Danish Data Protection Agency (registration number HGH-2017-039).

## Results

### Patient characteristics

In total, we audited health records of 2015 patients with CAP who were admitted at the study sites (Table 2). The cohort had a median age of 75 years and a balanced gender distribution. Chronic obstructive pulmonary disease (COPD) was the most common respiratory comorbidity among 30% of patients, followed by asthma in 9%, interstitial lung disease in 3%, lung cancer in 2% and bronchiectasis in 2%. Over half of all patients were former or current smokers; only 18% were never smokers. Smoking history was not documented in nearly one-third of the patient files.

**Table 2:** Patient characteristics.

Characteristics		All patients (n = 2015)	Site 1 (n = 694)	Site 2 (n = 532)	Site 3 (n = 242)	Site 4 (n = 547)
**Demographics**						
	Age in years, median (IQR)	75 (65, 84)	75 (64, 84)	79 (69, 88)	75 (68, 83)	72 (59, 82)
	Male sex, n (%)	981 (49)	340 (49)	257 (48)	113 (47)	271 (51)
**Respiratory comorbidities**, n (%)						
	COPD	602 (30)	193 (28)	120 (23)	94 (39)	195 (36)
	Asthma	186 (9)	68 (10)	49 (9)	13 (5)	56 (10)
	Bronchiectasis	47 (2)	11 (2)	11 (2)	15 (6)	10 (2)
	Lung cancer	51 (2)	5 (1)	13 (2)	13 (5)	20 (4)
	Interstitial lung disease	64 (3)	19 (3)	14 (3)	19 (8)	12 (2)
	Other	42 (2)	12 (2)	8 (2)	13 (5)	9 (2)
**Smoking history**, n (%)						
	Active smoker	355 (18)	76 (11)	86 (16)	41 (17)	152 (27)
	Former smoker	814 (40)	273 (39)	213 (40)	116 (47)	212 (39)
	Never smoker	313 (16)	126 (18)	61 (12)	33 (14)	93 (17)
	Not documented	533 (26)	219 (32)	172 (32)	52 (22)	90 (17)
**CURB-65**, n (%)						
	0	398 (20)	146 (21)	78 (15)	38 (15)	136 (25)
	1	701 (34)	243 (35)	170 (32)	87 (37)	201 (36)
	2	562 (29)	193 (28)	160 (30)	73 (30)	136 (25)
	3	283 (14)	88 (13)	98 (18)	39 (16)	58 (11)
	4	64 (3)	22 (3)	24 (5)	3 (1)	15 (3)
	5	7 (0)	2 (0)	2 (0)	2 (1)	1 (0)
**In-hospital death**, n (%)						
		143 (7)	48 (7)	41 (8)	11 (5)	43 (8)

Abbreviations: COPD: chronic obstructive pulmonary disease; IQR: interquartile range; CURB-65: pneumonia mortality risk score (confusion, urea, respiration frequency, blood pressure, age 65 or older) [32].

More than 50% of all patients had a CURB-65 score of 0 or 1, corresponding to a very low risk of pneumonia-related mortality [32]. Overall in-hospital mortality was 7% (range 5–8%). There were no statistically significant demographical differences between the sites.

### CAP care bundle utilisation over time

**Fig 2:**
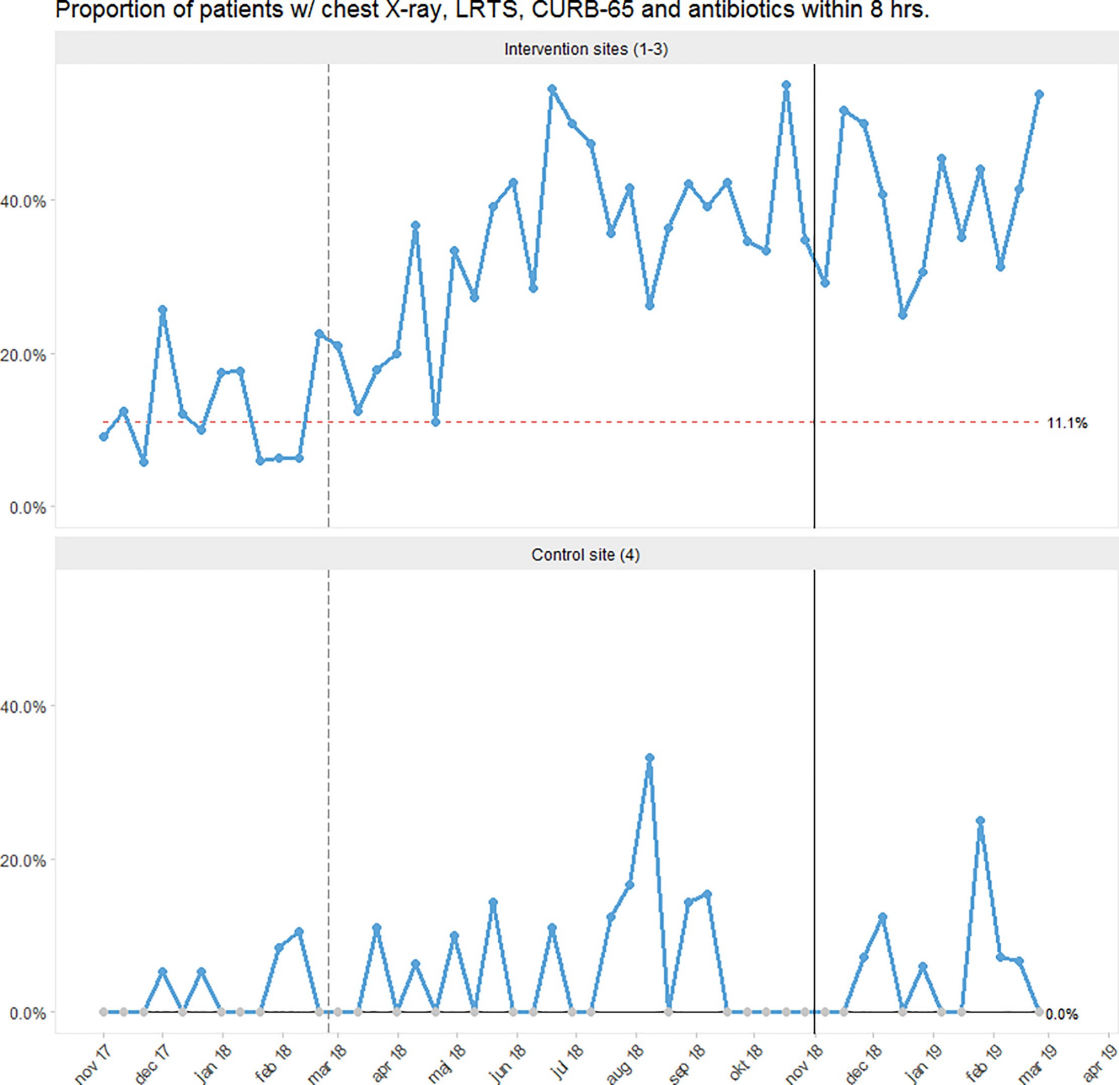
Run chart showing the proportion of patients receiving the CAP care bundle (i.e. chest X-ray, LRTS, CURB-65 and antibiotics) within 8 hours from admission. Each dot represents 12–48 cases of CAP. The vertical, grey, dashed line marks the beginning of the intervention period. The vertical, black, solid line denotes the beginning of the follow-up period. The process centre (horizontal line representing the median) was frozen after the baseline period. Special cause variation can be identified by a red, dashed process centre (sustained shift) [36]. See S2.1 Fig in S1 File for run charts for the individual intervention sites along with information on the timing of our interventions.

Since the beginning of the intervention period in March 2018, we noted a steady increase in care bundle utilisation, with special cause variation caused by a sustained shift at all the intervention sites (Fig 2, S2.1 and S2.2 Figs in S1 File) At the control site, we only observed random variation.

In the follow-up period, the care bundle was completed in 41% of cases as compared to 11% at baseline at the intervention sites, indicating sustained improvements (S2.2 Fig in S1 File). Meanwhile, the completion rate remained below 3% at the control site.

The detailed, site-specific run charts including information on the implementation schedule are presented in S2.1 Fig in S1 File.

### Utilisation of the individual elements of the care bundle over time

**Fig 3:**
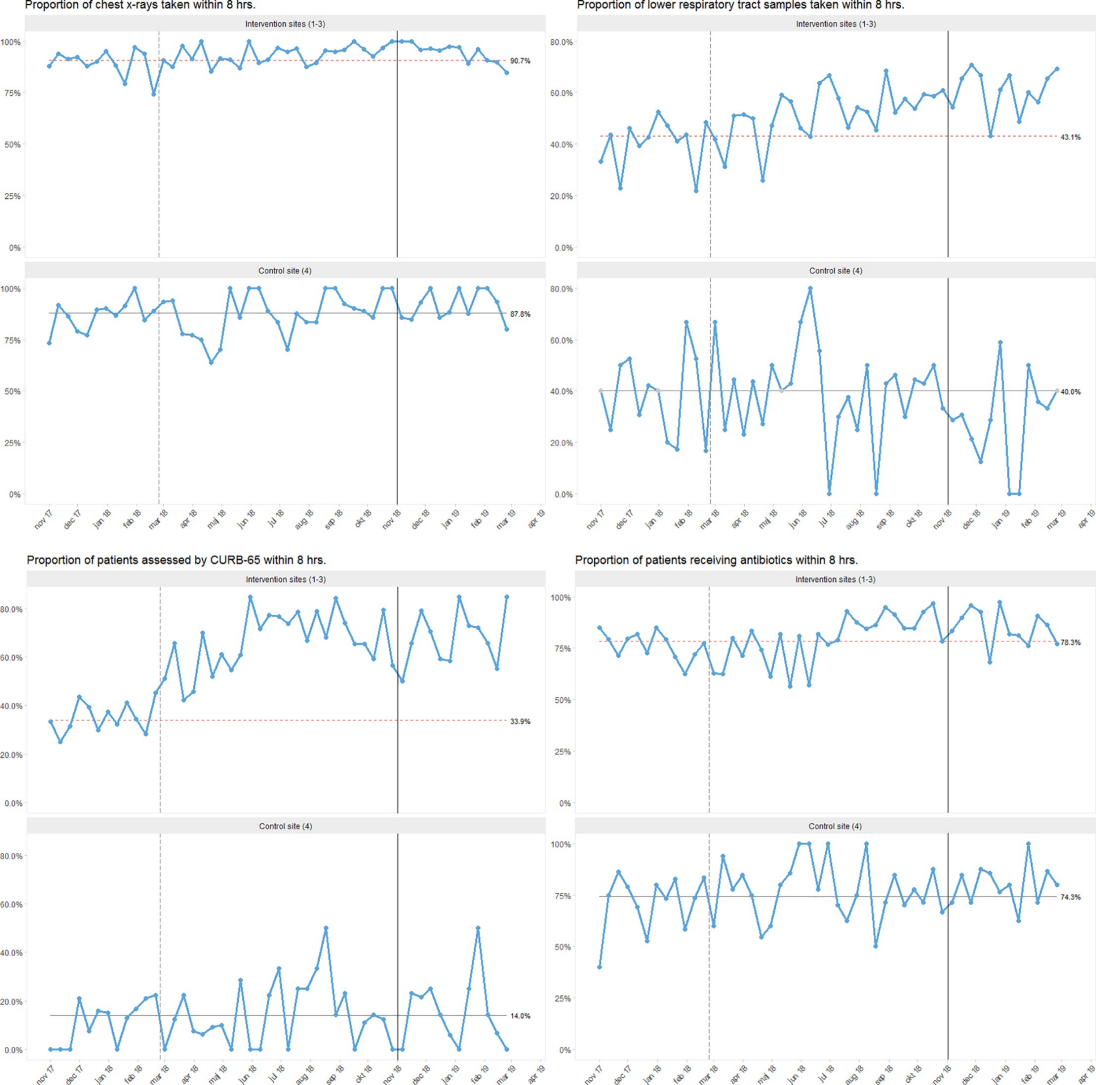
Run charts showing the proportion of patients receiving individual elements of the CAP care bundle within 8 hours from admission. Each dot represents 12–48 cases of CAP. The vertical, grey, dashed line denotes the beginning of the intervention period. The vertical, black, solid line marks the beginning of the follow-up period. The process centre (horizontal line representing the median) is frozen after the baseline period. Special cause variation can be identified by a red, dashed process centre (sustained shift) [36]. See S3.1–3.8 Figs in S1 File for run charts for the individual intervention sites along with information on the timing of our interventions.

At the intervention sites, we observed special cause variation for all the elements of the care bundle: chest X-ray, LRTS, CURB-65 score and antibiotics within 8 hours from admission (Fig 3). At the control site, we saw random variation without substantial improvements regarding all bundle elements throughout the study period.

#### Utilisation of chest X-ray within 8 hours

In general, all sites had a high utilisation rate of chest X-ray within 8 hours (S3.2 Fig in S1 File). While we observed a more stable process regarding the acquisition of X-rays within 8 hours at Site 3 during the baseline period, the other sites displayed more pronounced variation (S3.1 Fig in S1 File). At the intervention sites, we detected special cause variation after the end of the baseline period due to a sustained moderate shift, both in the run charts for the individual sites and the combined chart. Site 2 exhibited a more stable process towards 100% after authorizing nurses to order chest X-rays, the only intervention specifically targeting this element of the care bundle (S3.1 Fig in S1 File).

#### Acquisition of LRTS within 8 hours

Regarding the acquisition of LRTS, we observed special cause variation with an increase in LRTI samples taken within 8 hours at the intervention sites after baseline (Fig 3). The proportion of patients from whom LRTS were taken fluctuated and seemed to increase almost every time after performing the hands-on training in tracheal suction (S3.3 Fig in S1 File). The effect lasted about one month at the larger intervention sites (Sites 1 and 2). The proportion of LRTS taken improved from 43% in the baseline to 63% in the follow-up period at the intervention centres, whereas it decreased from 40% to 30% at the control centre (S3.4 Fig in S1 File).

#### Documentation of CURB-65 within 8 hours

The documentation of CURB-65 at baseline was generally low but highly variable amongst the study sites, ranging from 14% at Site 4 to 60% at Site 3. During the intervention period, a steady improvement in CURB-65 utilisation was observed at the intervention sites, whereas random variation continued at the control site (Fig 3). The most distinct improvements were seen at Sites 1 and 2 after the implementation of a CURB-65 tool for the EHRS (S3.5 Fig in S1 File). The documentation of CURB-65 increased from 34% in the baseline to 68% in the follow-up period at the intervention centres, while remaining stable at 14% at the control centre (S3.6 Fig in S1 File).

#### Administration of antibiotics within 8 hours

Regarding the final element of the care bundle—the administration of antibiotics within 8 hours from admission—we observed a high utilisation rate at all centres of 65% to 85% at baseline (S3.7 Fig in S1 File). Improvements with special cause variation after baseline were once again seen at the intervention sites, whereas random variation continued at the control site (Fig 3). The improvements were mainly driven by a moderate sustained shift at the larger intervention sites (Site 1 and 2; S3.7 Fig in S1 File).

## Discussion

### Main results

Our project aimed to improve patient care by increasing adherence to CAP guidelines through the implementation of tailored interventions. Throughout the study, changes in key process measures following the interventions were subsequently assessed and summarised in a CAP care bundle. Overall, we achieved sustained improvement in the use of the bundle comprised of chest X-ray, CURB-65, LRTS and antibiotics administered within 8 hours from hospital admission.

At baseline, completion rates for all defined indicators varied, and the study sites faced different challenges regarding the management of CAP patients. This information highlights the necessity of focusing on the local challenges when designing improvement studies, and to tailor the interventions to these challenges [27].

Only approximately one-third of all patients were scored with CURB-65 or had an LRTS taken within 8 hours from admission at the hospital. Antibiotics were administered within the recommended 8 hours in 75% of all cases. Chest X-ray within 8 hours was the only component that reached a completion rate of 89% at baseline.

Throughout the intervention period, we observed significant improvements with special cause variation at the intervention sites, especially regarding the documentation of CURB-65, but also in the acquisition of LRTS and, to a lesser degree, chest X-rays and antibiotic administration within 8 hours. The last two measures were those with the highest completion rates at the start of the study. Thus, it seems that the improvement potential was higher for the other two elements of the care bundle.

Our outcome measure was an all-or-none indicator, combining the key process measures in a care bundle [16]. Thus, the system we studied reflected the effectiveness of the health care system, as opposed to patient outcomes [27]. We chose this approach based on evidence from larger clinical studies clearly documenting that guideline-based treatment, also with the application of care bundles, improves outcomes in CAP [24,25,29,39–41].

Care bundle completion rates within 8 hours increased considerably at our intervention sites from 11% at baseline to 41% in the follow-up period.

The finding that only 41% of all CAP patients received assessments and treatments conforming to established guidelines might seem concerning. However, given that the care bundle relies on four independent elements, we believe that this result still is remarkable when compared to other studies on CAP care bundles reporting compliance rates of 20–29% [16,23,42]. LRTS collection can be challenging to improve in CAP, but it is regarded as crucial in the Nordics for potentially altering the often narrow-spectred empiric antibiotic treatment [4,30,31,43]. Omitting this element of the care bundle from our analysis yielded a completion rate of over 70%.

We used a range of interventions during the intervention period, all of which likely contributed to the observed improvements. We believe that the repeated educational activities with reiterations every 1–2 months were crucial for increasing and sustaining care bundle completion [44]. This is also reflected in the run charts, where compliance with the care bundle increased almost every time an educational activity took place. Beyond that, process changes, such as authorising nurses to order tests (i.e. chest X-rays and LRTS) and providing standard phrases and order sets in the EHRS to guide clinicians also seemed to be effective. In this context, a thorough review of local processes was vital to understanding the local difficulties and needs for advancement, as is generally recommended in quality improvement [27].

Since all of our interventions were reasonably simple, dedicated clinicians should be able to integrate them in their daily routine at little expense if they are given time to do so. Apart from human resources, the only intervention incurring additional costs was the pocket cards on CAP treatment.

The most time-consuming aspects of our project were the screening for CAP patients via the X-ray systems and the health record audit. Approximately 15 hours per week per site were spent on this task throughout the study period. We chose this approach because of the well-documented inaccuracy of the International Classification of Diseases (ICD)-10 diagnosis of pneumonia. In a recent Danish study, more than one fourth of patients diagnosed with pneumonia did not fulfil the criterion of a new infiltrate on chest X-ray [31].

To ensure a consistent and streamlined audit and reliable data across all sites, clinicians were not permitted to audit the health records. A simpler and more feasible audit approach should be implemented in a daily routine and could involve continuous monitoring of CAP cases by a team of dedicated clinical staff members.

### Strengths

The greatest strengths of our study are its size as well as the participation of one control and three intervention sites. The use of a control site is unusual in quality improvement, and the finding of improvements at the intervention sites together with a lack of change at the control site strengthens our conclusions [45]. In addition, we included a representative study population from study sites covering regions that serve approximately 15% of the Danish population. Hence, we think comparable programs could be designed throughout the country leading to comparable results.

Moreover, the study population was a real-life CAP cohort that is demographically comparable to another large Danish CAP cohort [31].

### Limitations

The main limitation of our study is the reliance on the audit of health records that only contain the information that health professionals document. We could not identify a study investigating the quality of documentation in the EHRS used at our study sites; however, based on our experiences, it is generally high. Nevertheless, we cannot be sure whether a certain proportion of patients did not meet the bundle care criteria due to a lack of documentation.

Finally, the limited follow-up period of only four months does not allow an assessment of sustainability. This question will be the focus of a follow-up study starting in November 2019 and continuing until February 2020.

## Conclusions

During the intervention period of our quality improvement project, we observed significant enhancement of patient care for CAP at the intervention sites with a simultaneous lack of improvement at the control site. Additionally, the standard of care remained much higher than at baseline during four months of follow-up. Our study emphasizes that the existence of guidelines alone does not assure high-quality patient care. We found that achieving improvements in CAP patient care required a combination of focus on the disease, locally tailored interventions and changes to key processes.

## Supporting information

S1 Data (CSV)

S2 Data (R)

S1 File (DOCX)
