# Supplementary material for: Improved treatment of community-acquired pneumonia through tailored interventions: Results from a controlled, multicentre quality improvement project
Source: PLoS One. 2020 Jun 11;15(6):e0234308. doi: 10.1371/journal.pone.0234308 (PMC7289425; doi:10.1371/journal.pone.0234308)

## Fig S1. Local context

**Fig S1. The local context and patient flow at the study sites.** Abbreviations: CAP: community-acquired pneumonia; ICU: intensive care unit.


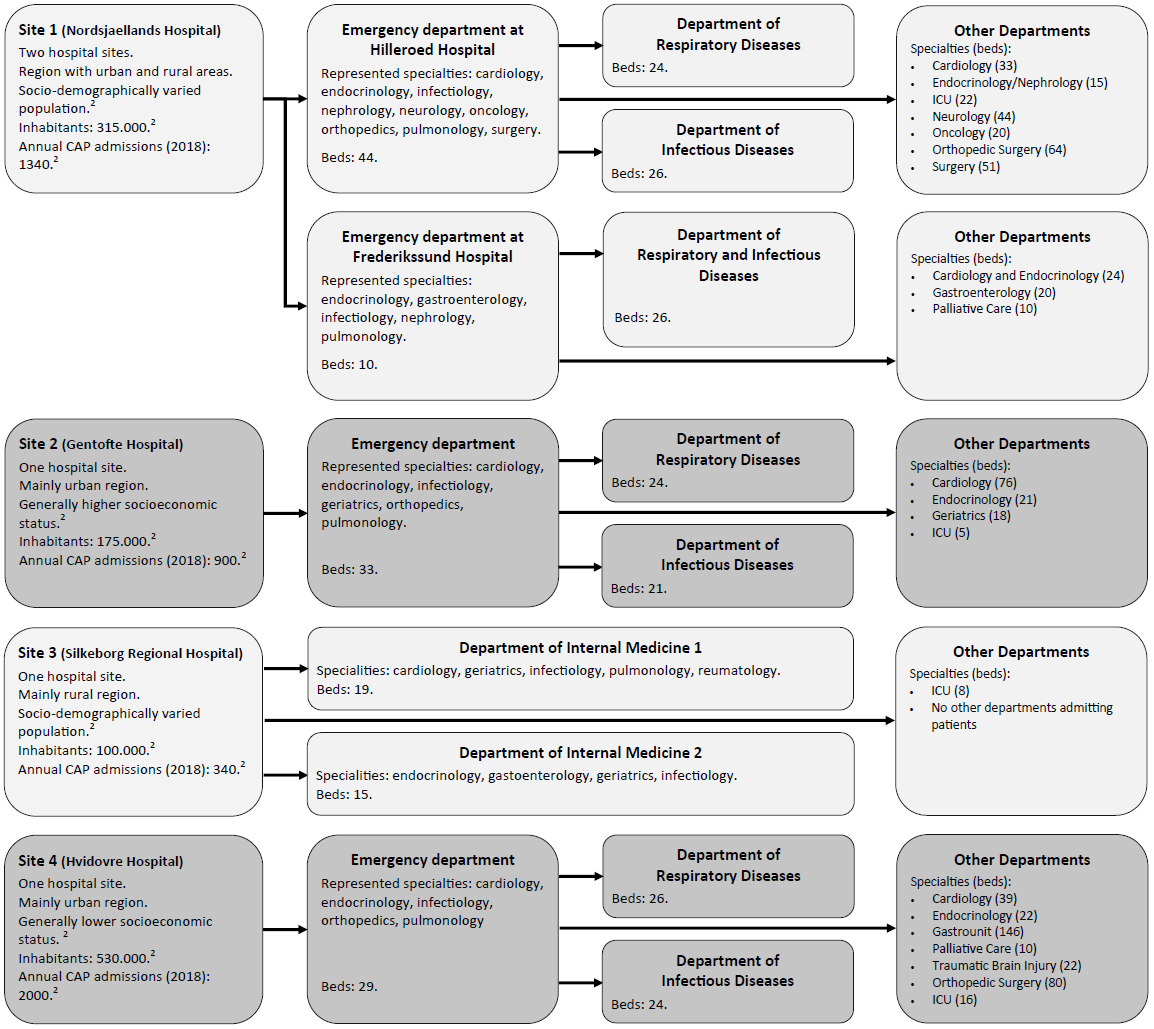


## S1. Details regarding the study sites

### Context Site 1 (Nordsjaellands Hospital)

This hospital, which is settled in the northern part of the Capital Region of Denmark, is comprised of two hospital sites (one in Hillerød and one in Frederikssund), as well as one healthcare centre in Helsingør. The hospital primarily serves the municipalities of Hillerød, Frederikssund, Fredensborg, Gribskov, Halsnæs, Helsingør, Allerød and Hørsholm, with a combined population of about 315.000.

There are emergency departments (EDs) at both hospital sites, which are open 24 hours a day in Hillerød and from 8 a.m. to 4 p.m. in Frederikssund. Often, patients admitted to the ED at Hillerød are being transported to Frederikssund when there is a need for hospitalisation but no need for an intensive care unit. Patients included in optiCAP were all initially admitted at the ED in Hillerød.

The ED at Hillerød is led by a head consultant and head nurse. Overall, approximately 100 nurses are employed in this department; however, only a few physicians are employed directly at the ED. Most physicians treating patients in the ED are employed various speciality departments, which are also responsible for organising the shifts for physicians.

The ED, with its 44 beds, is comprised of four bases and one triage situated next to each other on one floor. All patients are registered by a secretary at arrival and are thereafter seen by a nurse in the triage, where the patient’s condition is evaluated, and further treatment and assessment is planned. Patients are then transported to one of the bases, where they are seen by a physician working in the respective specialty:

- Base 1: cardiology, nephrology, endocrinology
- Base 2: respiratory medicine, infectious diseases, neurology, oncology
- Base 3: orthopaedic surgery
- Base 4: surgery

Patients with pneumonia are usually admitted in base 2 and only these patients were included in optiCAP. Some patients are handled at the ED only, while others are admitted to the different specialised departments. If patients with pneumonia are transferred from the ED, they are usually admitted at the Departments for Respiratory Medicine (24 beds, approximately 27 nurses) or Infectious Diseases (26 beds, approximately 26 nurses) or they are transferred to the Department for Respiratory Medicine and Infectious Diseases at Frederikssund Hospital (26 beds, approximately 23 nurses).

### Context Site 2 (Gentofte Hospital)

Gentofte Hospital is the smaller of two hospital sites in the western part of the Capital Region of Denmark, consolidated as Herlev Gentofte Hospital. The hospital primarily serves the municipalities of Gentofte, Furesø, Lyngby-Taarbæk, and Rudersdal, with a combined population of approximately 175.000.

The ED is open 24 hours a day for patients admitted by the Copenhagen Emergency Medical Services to one of the departments for internal medicine, and from 7 a.m. to 10 p.m. for patients with injuries.

Both EDs (in Gentofte and Herlev) are under the same administration and led by a head consultant and a head nurse. All in all, approximately 40 nurses are employed in this department. All physicians working at the ED in Gentofte are employed at the various speciality departments, which are also responsible for organising the shifts for physicians.

The ED in Gentofte, with its 33 beds, is divided into two sections where different patients are handled:

- Section 201 (on the ground floor): cardiology, orthopaedic surgery, internal medicine
- Section 211 (on the first floor): only patients with the need for hospitalisation at a department for internal medicine

All patients must leave the ED within 24 hours, unless the patient is too unstable to be handled at the specialty departments and has the need for intermediate care (two beds in Section 211 are dedicated for this purpose). If further hospitalisation is required for stable patients, this must occur at one of the specialised departments at either Gentofte (Respiratory Medicine, Infectious Diseases, Cardiology, Endocrinology or Geriatrics) or Herlev Hospital (all specialities). Patients with pneumonia are usually admitted at the Departments for Respiratory Medicine (24 beds, approximately 15 physicians and 25 nurses) or Infectious Diseases (21 beds, approximately 8 physicians and 22 nurses).

### Context Site 3 (Silkeborg Regional Hospital)

Silkeborg Regional Hospital is one of two major hospitals of a hospital unit in the Central Denmark Region - the other one being Viborg Regional Hospital. Silkeborg Regional Hospital serves a population of approximately 100.000.

At Silkeborg Regional Hospital, there is no ED, but patients with medical problems may be directly admitted via the region’s visitation unit or an outpatient clinic to the Diagnostic Centre on working days between 8 a.m. and 5 p.m. Between 5 p.m. and 8 a.m. and on weekends, only certain patients are admitted, such as patients transferred from the ED at Viborg Regional Hospital or patients with an open admission (i.e. frequent admissions at the department).

The Diagnostic Centre (DC) at Silkeborg Regional Hospital is comprised of two medical wards (M1 and M2) and a radiology department under the same administration. Approximately 95 physicians are employed at the DC. Of these, 75 work in the medical wards. Patients with pneumonia may either be admitted at ward M1 (15-19 beds, approximately 65 nurses) or ward M2 (15 beds, approximately 30 nurses).

The following specialities are included in the following medical wards:

- M1: respiratory medicine, infectious diseases, geriatrics, cardiology, rheumatology
- M2: infectious diseases, endocrinology, gastroenterology, geriatrics

### Context Site 4 (Hvidovre Hospital)

Hvidovre Hospital is situated in the southern part of the Capital Region of Denmark and includes a smaller hospital site at Amager. Together, these sites serve a population of approximately 530.000 in the municipalities of Copenhagen, Høje-Taastrup, Albertslund, Ishøj, Vallensbæk, Brøndby, Glostrup, Hvidovre, Tårnby and Dragør.

The ED at Hvidovre Hospital is available 24 hours a day and has 29 beds. The ED is led by a head consultant and a head nurse. All patients are registered by a secretary at arrival and are then seen by a nurse in the triage, where the patient’s condition is evaluated, and further treatment and assessment is planned. Over 100 nurses and <10 physicians are employed directly within the ED. Otherwise, physicians working in the ED are employed at the Internal Medicine Departments. If patients admitted to the ED require hospitalisation beyond 24-48 hours, they are moved to available beds in other wards.

Patients hospitalised with pneumonia are primarily transferred to the Department of Infectious Diseases (24 beds, approx. 36 nurses, 20 physicians) or the Department of Respiratory Disease (26 beds, approx. 30 nurses, 24 physicians). The latter forms part of the Department of Internal Medicine, which additionally comprises the Departments of Cardiology and Endocrinology and is led by the same head consultant and head nurse.

## Figs S2.1 and S2.2 showing bundle care delivery

**Fig S2.1. Run chart showing bundle care delivery within 8 hours together with the specific interventions.** The vertical, grey, dashed line marks the beginning of the intervention period. The process centre (horizontal line representing the median) is frozen after the baseline period. Special cause variation can be identified by a red, dashed process centre (sustained shift).

**
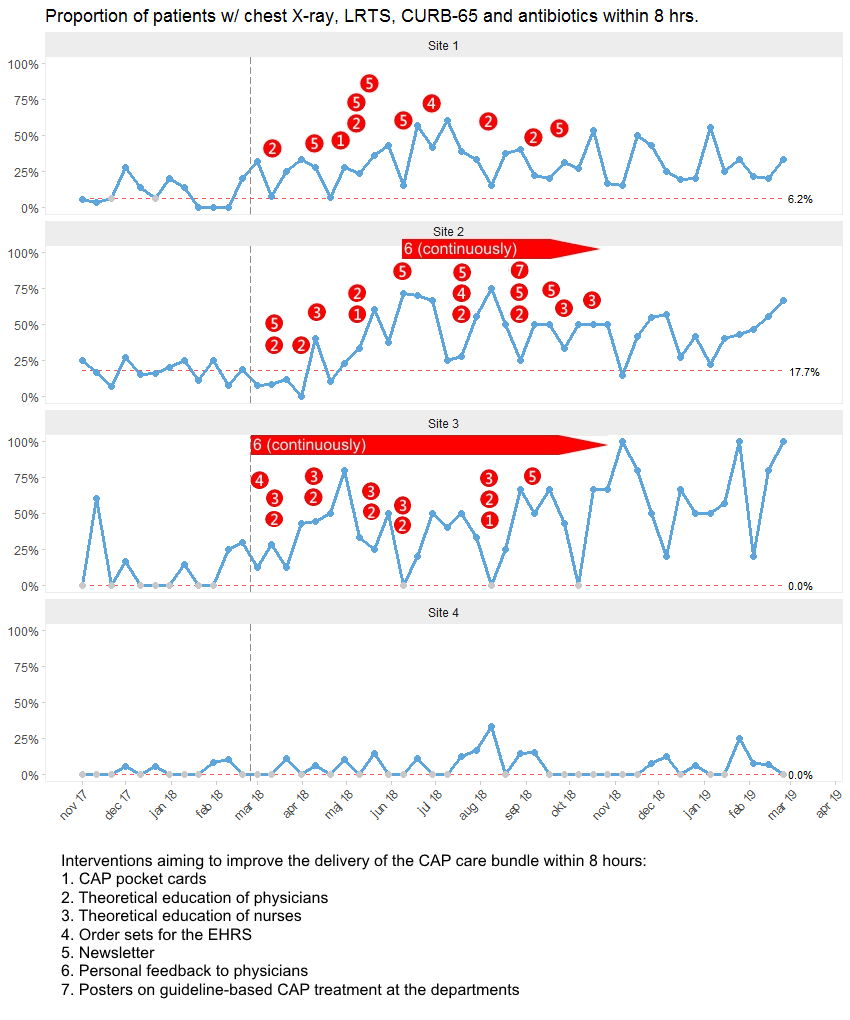
**

**Fig S2.2. Run chart showing bundle care delivery within 8 hours.** The vertical, grey, dashed line marks the beginning of the intervention period. The process centre (horizontal line representing the median) has been recalculated for four periods of four months each (baseline, early intervention, late intervention and follow-up).

**
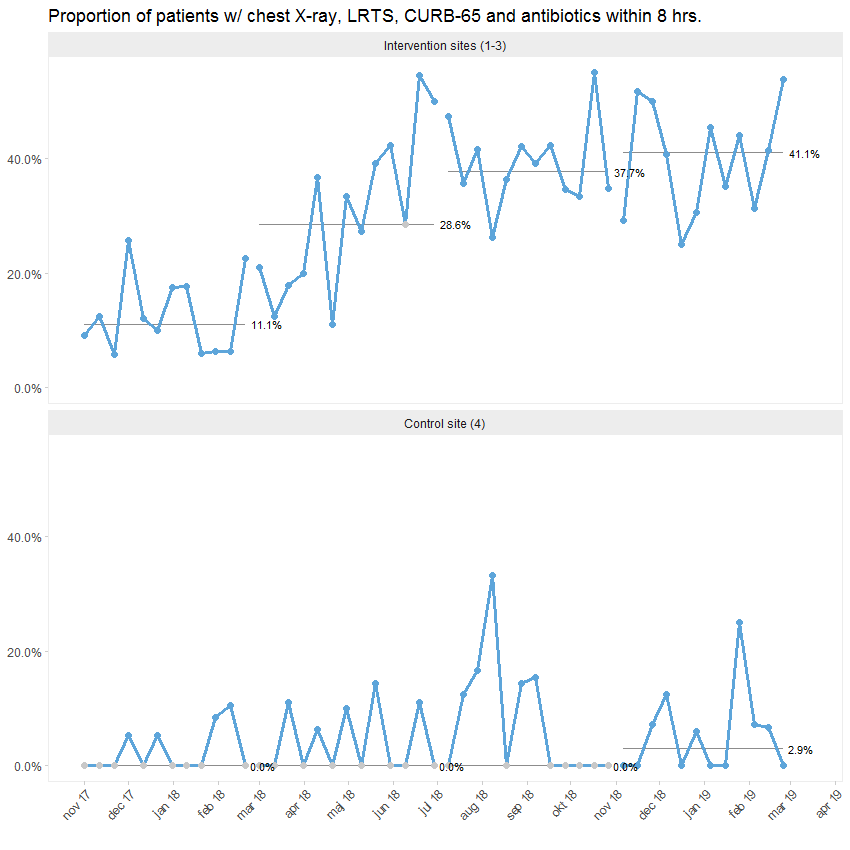
**

## Figs S3.1-S3.8 showing delivery of the individual elements of the care bundle

**Fig S3.1. Run chart showing the proportion of chest X-rays done within 8 hours together with the specific interventions.** The vertical, grey, dashed line marks the beginning of the intervention period. The process centre (horizontal line representing the median) is frozen after the baseline period. Special cause variation can be identified by a red, dashed process centre (sustained shift).

**
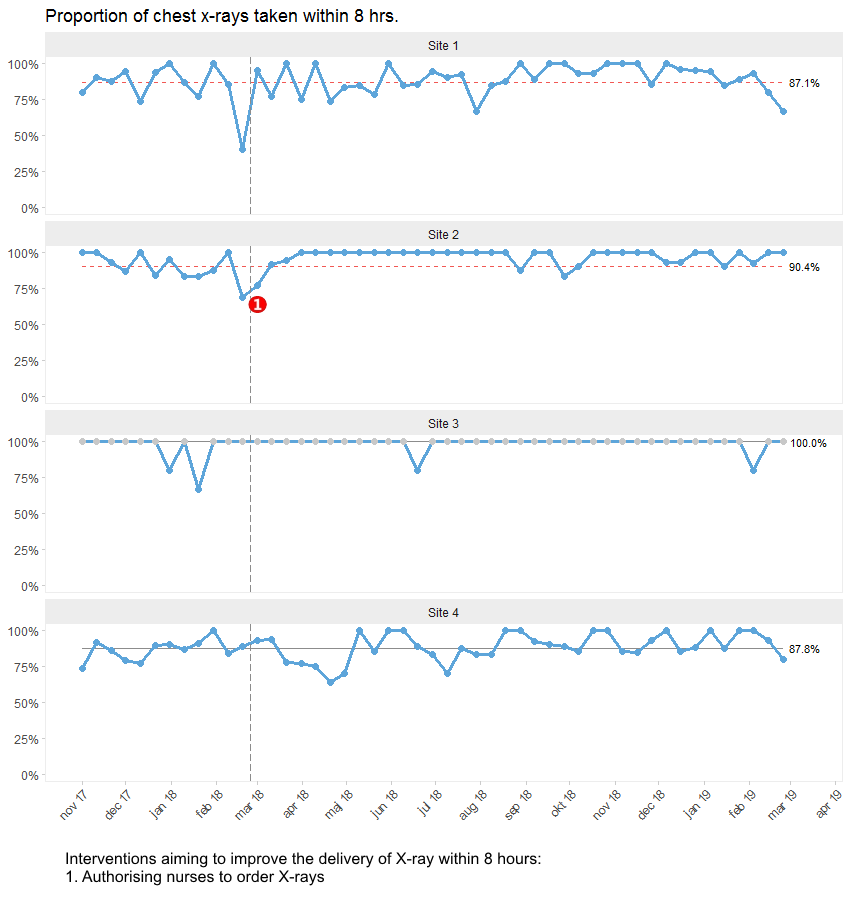
**

**Fig S3.2. Run chart showing the proportion of chest X-rays done within 8 hours.** The vertical, grey, dashed line marks the beginning of the intervention period. The process centre (horizontal line representing the median) has been recalculated for four periods of four months each (baseline, early intervention, late intervention and follow-up).


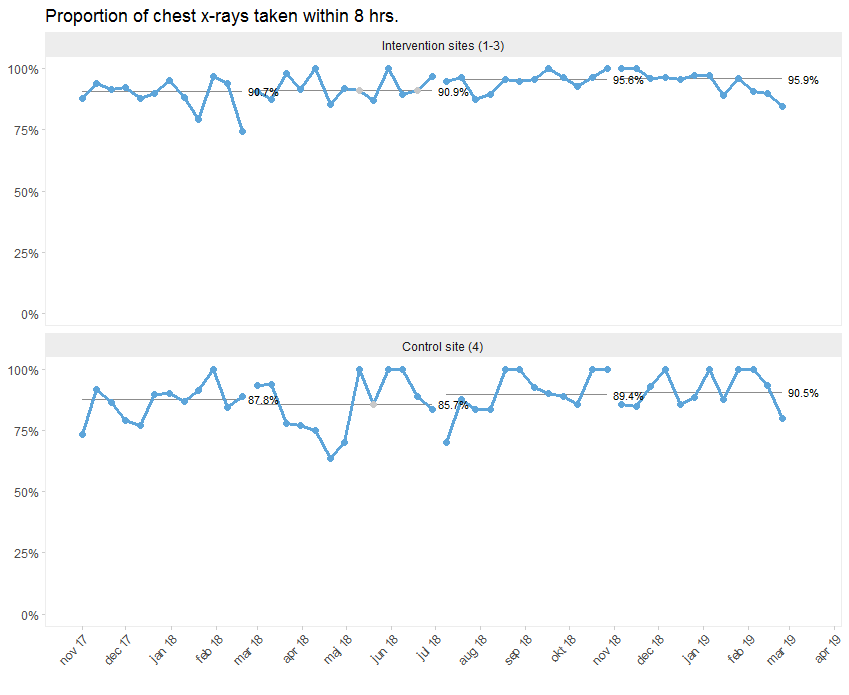


**Fig S3.3. Run chart showing the proportion of LRTS done within 8 hours together with the specific interventions.** The vertical, grey, dashed line marks the beginning of the intervention period. The process centre (horizontal line representing the median) is frozen after the baseline period. Special cause variation can be identified by a red, dashed process centre (sustained shift).

**
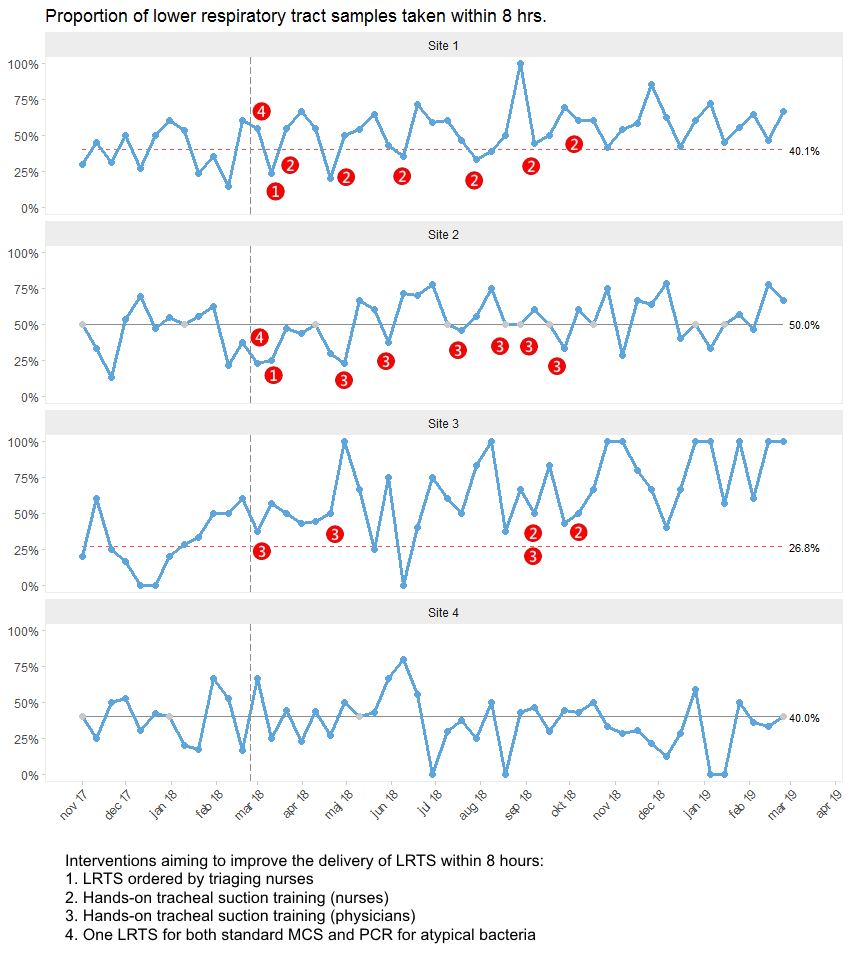
**

**Fig S3.4. Run chart showing the proportion of LRTS done within 8 hours.** The vertical, grey, dashed line marks the beginning of the intervention period. The process centre (horizontal line representing the median) has been recalculated for four periods of four months each (baseline, early intervention, late intervention and follow-up).

**
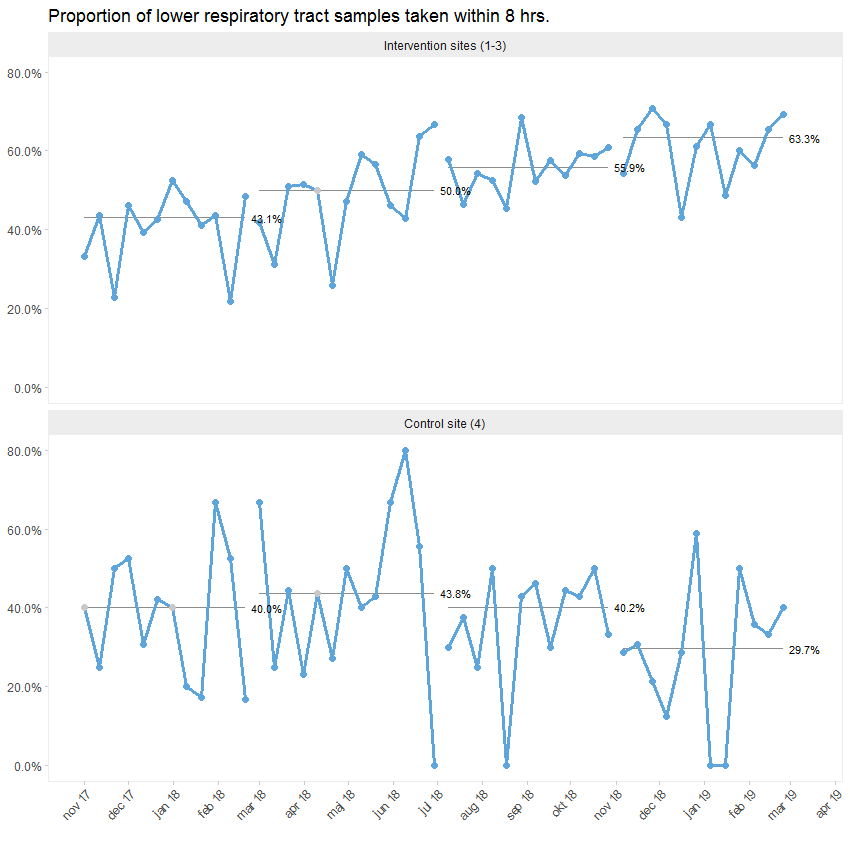
**

**Fig S3.5. Run chart showing the proportion CURB-65 documented within 8 hours together with the specific interventions.** The vertical, grey, dashed line marks the beginning of the intervention period. The process centre (horizontal line representing the median) is frozen after the baseline period. Special cause variation can be identified by a red, dashed process centre (sustained shift).

**
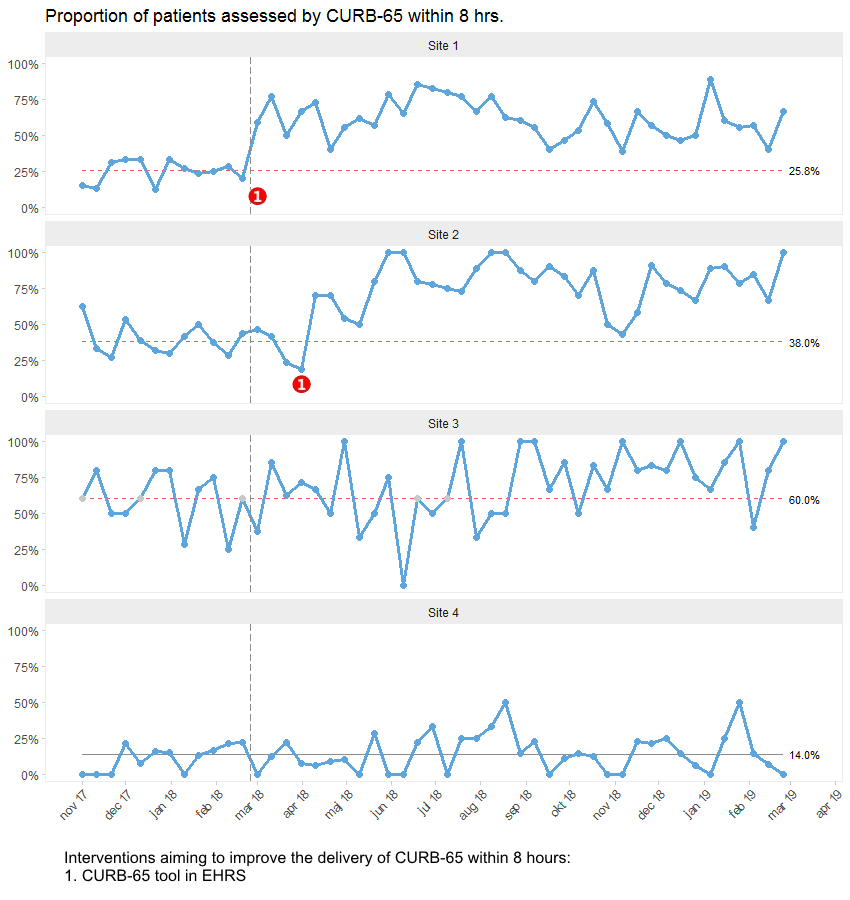
**

**Fig S3.6. Run chart showing the proportion of CURB-65 documented within 8 hours.** The vertical, grey, dashed line marks the beginning of the intervention period. The process centre (horizontal line representing the median) has been recalculated for four periods of four months each (baseline, early intervention, late intervention and follow-up).


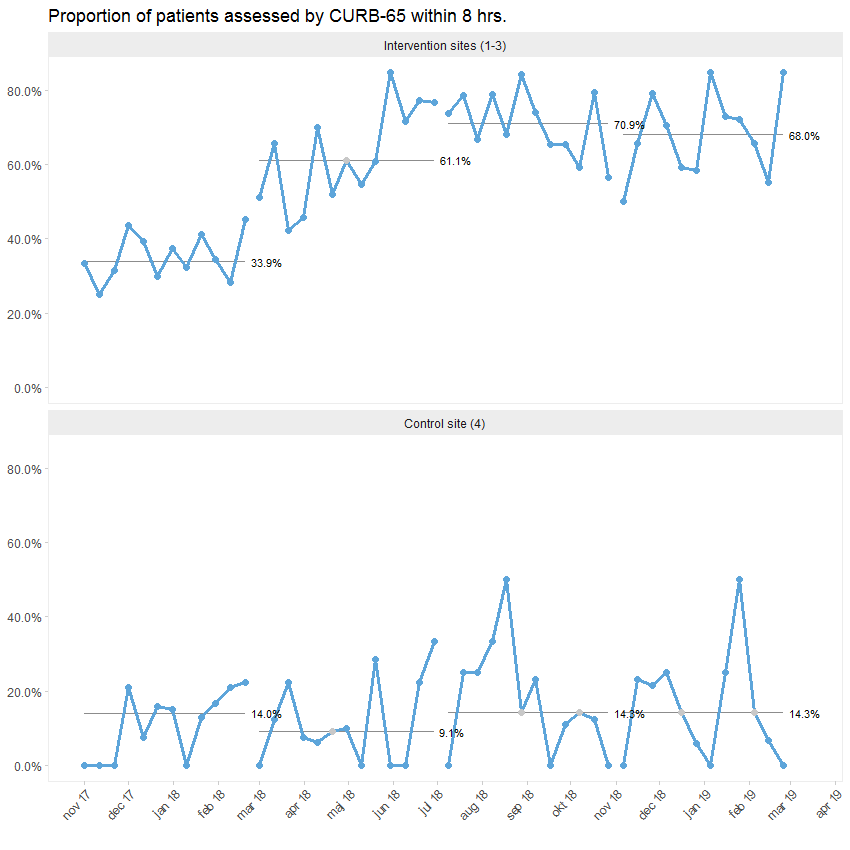


**Fig S3.7. Run chart showing the proportion of patients receiving antibiotics within 8 hours.** The vertical, grey, dashed line marks the beginning of the intervention period. The process centre (horizontal line representing the median) is frozen after the baseline period. Special cause variation can be identified by a red, dashed process centre (sustained shift).

**
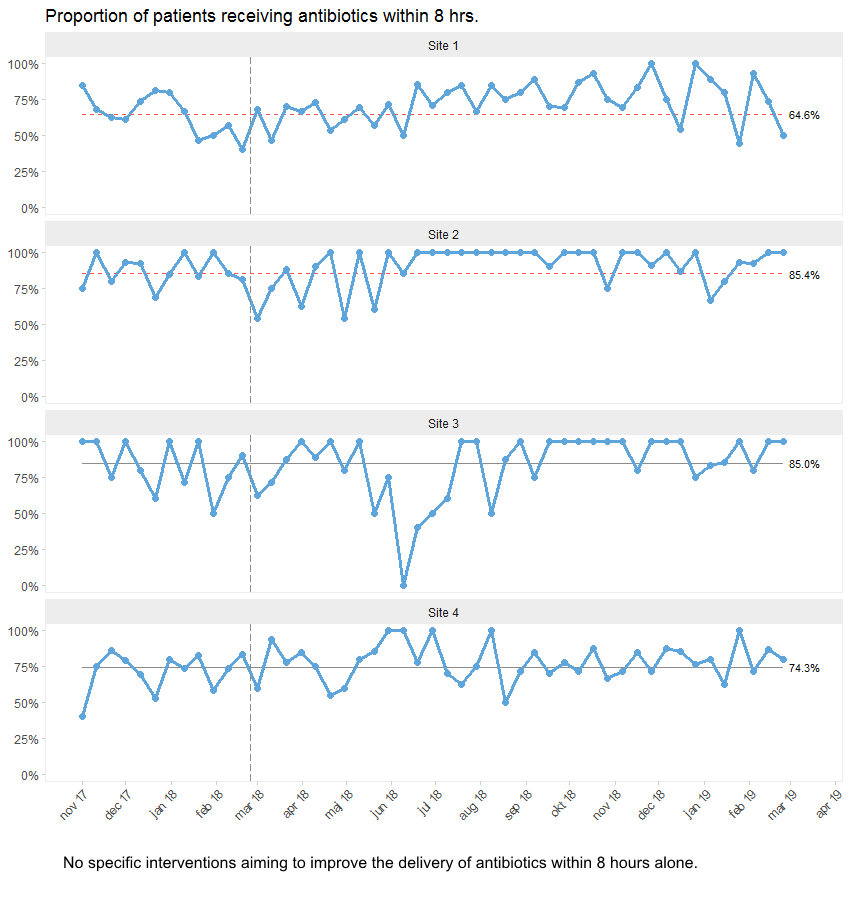
**

**Fig S3.8. Run chart showing the proportion of patients receiving antibiotics within 8 hours.** The vertical, grey, dashed line marks the beginning of the intervention period. The process centre (horizontal line representing the median) has been recalculated for four periods of four months each (baseline, early intervention, late intervention and follow-up).


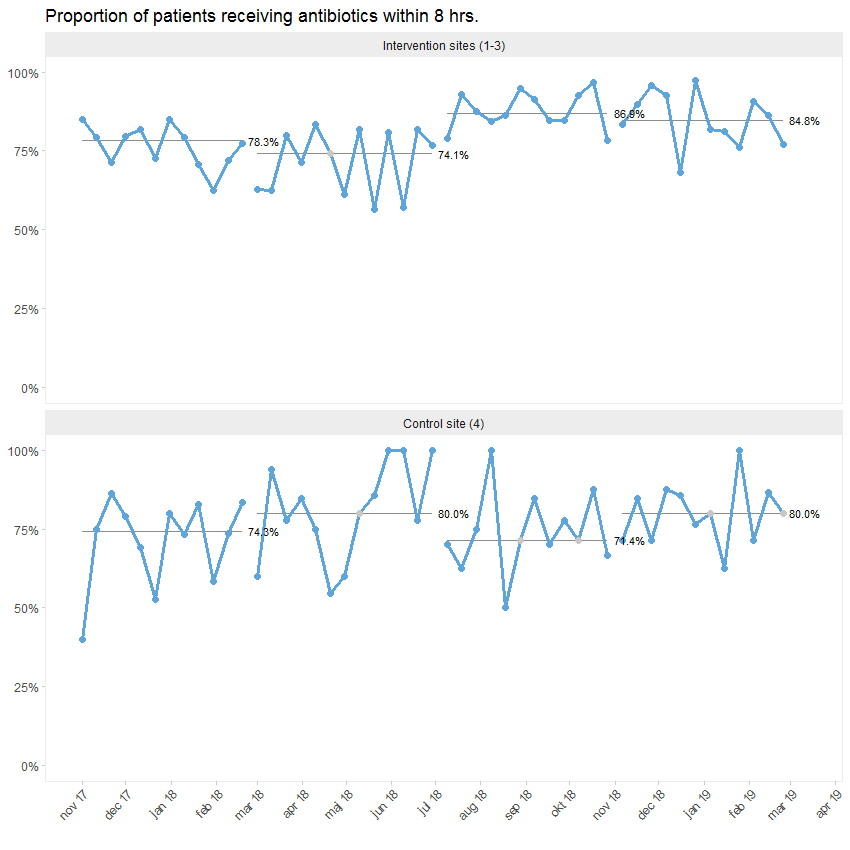

Supplement: S1 File — (DOCX) [file pone.0234308.s003.docx]
